# Remarkable Iodine-Catalyzed Synthesis of Novel Pyrrole- Bearing *N*‑Polyaromatic β-Lactams 

**DOI:** 10.3390/molecules15021082

**Published:** 2010-02-23

**Authors:** Debasish Bandyopadhyay, Gildardo Rivera, Isabel Salinas, Hector Aguilar, Bimal K. Banik

**Affiliations:** Department of Chemistry, The University of Texas-Pan American, 1201 West University Drive, Edinburg, TX 78541, USA

**Keywords:** iodine, catalysis, β-lactams, pyrroles

## Abstract

Because of their interesting biological properties various methods for the synthesis of substituted pyrroles are described in the literature. However, synthesis of pyrroles fused with a β-lactam ring has not been reported. Our group has demonstrated synthesis and biological evaluation of various β-lactams as anticancer agents. The anticancer activities of these compounds have prompted us to study the synthesis of pyrroles bound to the β-lactams. We have identified an expeditious synthetic method for the preparation of pyrroles fused with β-lactams by reacting 3-amino β-lactams with acetonylacetone in the presence of catalytic amounts (5 mol%) of molecular iodine at room temperature. It has also been discovered that the reaction gives products under domestic and automated microwave oven irradiation. To our knowledge, there are no other prior reports that describe the synthesis of pyrrole-substituted β-lactams.

## 1. Introduction

The biological activities of pyrroles have investigated extensively [1,2]. For this reason, diverse methods for the synthesis of pyrroles are described in the literature [3]. Conjugate addition reactions have been performed for the preparation of polysubstituted pyrroles [4]. Pyrroles have also been prepared from transition metal intermediates [5], reductive coupling reactions [6], aza-Wittig reactions [7], and other multi-step processes [8,9]. Despite these new developments, the Paal-Knorr [10] reaction has remained one of the most attractive methods for the synthesis of pyrroles. Clay-induced [11] reactions and a microwave irradiation method [12] have been used for the synthesis of pyrroles under Paal-Knorr conditions. Reactions in the presence of silica gel and alumina as solid supports have been investigated, but the reactions did not proceeded to completion. The use of montmorillonite clay under identical conditions, however, has produced products in good yield. These methods have extended the scope of pyrrole synthesis, although synthesis of pyrroles attached to a β-lactam ring has not been reported. In this paper, we describe a simple and rapid iodine-catalyzed method for the synthesis of substituted pyrroles bound to several racemic *trans*-β-lactams. 

## 2. Results and Discussion

Our group has reported the synthesis and biological evaluation as novel anticancer agents of various β-lactams and polyaromatic compounds [13,14,15,16,17]. It was reported that alteration of the heterocyclic ring is determining in the biological activity of these compounds. The anticancer activity of these derivatives had prompted us to undertake an exploratory program in the synthesis of pyrroles bound to β-lactams with different structures. Our laboratory is engaged in devising synthetic methods which are practical or ecologically friendly [18,19,20,21,22,23,24,25]. The reaction between **1** and **2** does not proceed at all without iodine even after 10 minutes of microwave irradiation. The presence of small amounts of iodine (5 mol%) is necessary for the success of the reaction. The same has been studied extensively in our laboratory in several iodine-catalyzed reactions [26,27,28,29,30,31]. 

**Scheme 1:**
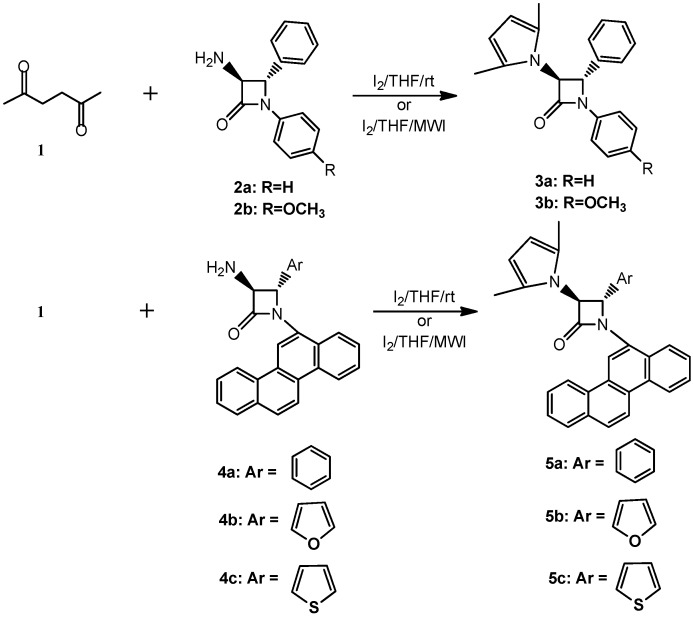
Synthesis of 3-pyrrole substituted β-lactams.

In continuation of our studies on iodine-catalyzed reactions [29,30,31], we have identified an expeditious synthetic method for the preparation of pyrroles fused with β-lactams starting from 3-amino β-lactams and 1,4-diketo compound at room temperature and using microwave-induced reactions. To our knowledge, there are no other prior reports that describe the iodine-catalyzed synthesis of pyrrole-substituted β-lactams. Several *trans* 3-amino β-lactams were used for this study. The other starting material was commercially available 2,5-hexanedione (Scheme 1).

The diketo compound, the amine and iodine (catalytic amounts) were mixed in ethanol and stirred overnight at room temperature. Upon extraction of the reaction mixture, 80–90% products were obtained. In another method, the mixture was irradiated in a CEM microwave oven for 5 min at a power level of 300 µ power. The reaction mixture was then extracted with dichloromethane. The reaction proceeded equally well irrespective of the nature of substituent present in the β-lactam ring. The iodine-catalyzed pyrrole formation reaction of 3-amino β-lactams (±)**-2** and (±)**-4** as described herein is very simple and can be used with remarkable success. In view of our earlier publications on anticancer β-lactams, this method may find future applications.

## 3. Experimental

### 3.1. General

Melting points were measured in a Fisher Scientific electrochemical Mel-Temp* manual melting point apparatus (Model 1001) equipped with a 300°C thermometer. Elemental analysis (C, H, N) were conducted using the Perkin-Elmer 2400 series II elemental analyzer, their results were found to be in good agreement (± 0.2%) with the calculated values for C, H, N. FT-IR spectra were registered on a Bruker IFS 55 Equinox FTIR spectrophotometer as KBr discs. ^1^H-NMR (300 MHz) and ^13^C-NMR (75.4 MHz) spectra were obtained at room temperature with JEOL Eclipse-300 equipment using TMS as internal standard and CDCl_3_ as solvent. Analytical grade chemicals (Sigma-Aldrich incorporation) were used throughout the project. Deionized water was used for the preparation of all aqueous solutions.

#### 3.1.1. General procedure for the synthesis of imines

Amine and aldehyde were mixed in equimolar ratio and refluxed in dry toluene using a Dean-Stark water separator. After completion of the reaction (monitored by TLC), the solvent was removed by rotavapor and the pure imine was isolated by crystallization from diethyl ether/hexanes. Polyaromatic amine (6-amino chrysene) took longer time than their monoaromatic analogues. 

#### 3.1.2. General procedure for the synthesis of 3-phthalimido β-lactams

In dry dichloromethane (5 mL), phthalimidoacetic acid (1.5 mmol) was mixed with 2-chloro-*N*-methylpyridinium iodide (3 mmol) and dry triethylamine (6 mmol) at 0 °C and the mixture was stirred for two hours. The imine (1 mmol) in dry dichloromethane (5 mL) was added drop wise and stirring was continued for another two hours at 0 °C to room temperature. After that, the mixture was refluxed for 14 hours (monitored by TLC) to obtain a single 3-phthalimido-substituted *trans* β-lactam. The reaction mixture was washed with saturated aqueous solution of sodium bicarbonate, brine and water successively. The organic layer was dried over anhydrous sodium sulfate and crystallized from dichloromethane/hexanes following usual procedure. The overall isolated yield was 76-85% [32]. The reaction proceeded well with diverse imines that have different types of aromatic groups.

#### 3.1.3. General procedure for the synthesis of 3-amino β-lactams

3-Phthalimidoβ-lactam (1 mmol) was mixed with ethylenediamine (1.5 mmol) in dry ethanol (3 mL) and stirred at room temperature for 15 minutes (monitored by TLC). After completion of the reaction, the reaction mixture was washed with brine and water successively. The organic layer was dried over anhydrous sodium sulfate and the 3-amino β-lactam was isolated in pure form by column chromatography over neutral alumina (methanolic ethyl acetate) in good yield [33]. The stereochemistry of β-lactams remains unchanged during this conversation. No cleavage of theβ-lactam rings was observed under this condition. 

### 3.2. General procedure for the synthesis of pyrroles *(±)-**3*** and *(±)-**5***

To a solution of the β-lactam amine (±)**2** or (±)**4** (1 mmol) and hexane-2,4-dione (**1, **1.2 mmol) in ethanol (1 mL) at room temperature, iodine (5 mol%) was added. The mixture was irradiated using a CEM DISCOVER Microwave at 300 µ power. Dichloromethane (10 mL) was added to the reaction mixture and it was then washed successively with 5% NaHCO_3_ solution (2 mL) and brine (2 mL) and water (2 mL). The organic layer was dried with sodium sulfate and concentrated. These pyrroles (±) **3** or (±)**5** were found to be sufficiently pure (more than 90%) and finally crystallized from dichloromethane/hexane.

*3-(2,5-Dimethyl-1H-pyrrol-1-yl)-1,4-diphenylazetidin-2-one* (±)**3a**. White crystals. Crystallized from dichloromethane/hexane mixture; Mp: 158 °C; IR: 2915, 1754, 1312, 1108, 780, 612 cm^-1^; ^1^H-NMR δ (ppm): 2.08 (s, 6H, CH_3_), 5.01 (d, *J* = 2.46, H-4), 5.13 (d, *J* = 2.49, H-3), 5.78 (s, 2H, pyrrole), 7.26-7.50 (m, 10H, Ar-H); ^13^C-NMR δ (ppm): 13.05 (2C), 64.05, 70.19, 107.51 (2C), 117.55 (2C), 124.76, 125.77 (2C), 128.66, 129.06 (2C), 129.37 (2C), 129.53 (2C), 136.02, 137.08, 163.67. Anal. Calcd for C_21_H_20_N_2_O: C, 79.72; H, 6.37; N, 8.85. Experimental: C, 79.68; H, 6.31; N, 8.77.

*3-(2,5-Dimethyl-1H-pyrrol-1-yl)-1-(4-methoxyphenyl)-4-phenylazetidin-2-one* (±)**3b**. Brown crystals. Crystallized from dichloromethane/hexane mixture; Mp: 149 °C; IR: 2922, 1752, 1297, 1110, 776 cm^-1^; ^1^H-NMR δ (ppm): 2.14 (s, 6H, CH_3_), 3.72 (s, 3H, OCH_3_), 5.14 (d, *J* = 2.31, H-4), 5.25 (d, *J* = 2.31, H-3), 5.71 (s, 2H, pyrrole), 7.25-7.81(m, 9H, Ar-H); ^13^C-NMR δ (ppm): 13.09 (2C), 55.52 (OCH_3_), 64.71, 66.89, 107.90 (2C), 117.87 (2C), 119.71 (2C), 126.04, 127.65 (2C), 128.32 (2C), 128.74 (2C), 132.50, 143.76, 158.43, 166.36. Anal. Calcd for C_22_H_22_N_2_O_2_: C, 76.28; H, 6.40; N, 8.09. Experimental: C, 76.14; H, 6.36; N, 8.02.

*1-(Chrysen-6-yl)-3-(2,5-dimethyl-1H-pyrrol-1-yl)-4-phenylazetidin-2-one* (±)**5a**. Pale yellow crystals. Crystallized from dichloromethane/hexane mixture; Mp: 111―113 °C; IR: 2920, 1754, 1302, 1112, 780 cm^-1^; ^1^H-NMR δ (ppm): 2.24 (s, 6H, CH_3_), 5.44 (d, *J* = 2.19, H-4), 5.67 (d, *J* = 2.19, H-3), 5.88 (d, *J* = 1.11, 2H, pyrrole), 7.24-8.12 (m, 16H, Ar-H); ^13^C-NMR δ (ppm): 13.41(2C), 65.73, 68.47, 107.71 (2C), 114.07, 122.87, 126.04 (2C), 126.09 (2C), 127.04 (2C), 128.45 (2C), 127.62 (2C), 128.74 (2C), 129.07 (2C), 129.38 (2C), 129.97 (2C), 131.19(2C), 131.98, 132.50, 135.90, 148.50, 165.05. Anal. Calcd for C_33_H_26_N_2_O: C, 84.95; H, 5.62; N, 6.00. Experimental: C, 84.86; H, 5.59; N, 5.93.

*1-(Chrysen-6-yl)-3-(2,5-dimethyl-1H-pyrrol-1-yl)-4-(furan-2-yl)azetidin-2-one* (±)**5b**. Yellow crystals. Crystallized from dichloromethane/hexane mixture; Mp: 135―137 °C; IR: 2912, 1753, 1310, 1110, 782 cm^-1^; ^1^H-NMR δ (ppm): 2.30 (s, 6H, CH_3_), 5.47 (d, *J* = 2.46, H-4), 5.65 (d, *J* = 2.46, H-3), 5.91 (s, 2H, pyrrole), 6.77 (m, 2H, furan), 7.35-8.59 (m, 12H, Ar-H); ^13^C-NMR δ (ppm): 13.45(2C), 65.63, 68.55, 107.65 (2C), 114.41, 114.77 (2C), 122.98, 123.77 (2C), 124.56, 126.86,127.00 (2C), 127.55 (2C), 127.81(2C), 128.78 (2C), 129.07 (2C), 129.38 (2C), 129.97, 135.98, 141.05, 160.14, 165.23. Anal. Calcd for C_31_H_24_N_2_O_2_: C, 81.56; H, 5.30; N, 6.14. Experimental: C, 81.58; H, 5.29; N, 6.08.

*1-(Chrysen-6-yl)-3-(2,5-dimethyl-1H-pyrrol-1-yl)-4-(thiophen-2-yl)azetidin-2-one* (±)**5c**. Yellowish white crystals. Crystallized from dichloromethane/hexane mixture; Mp: 231―233 °C; IR: 2914, 1755, 1306, 1105, 784 cm^-1^; ^1^H-NMR δ (ppm): 2.34 (s, 6H, CH_3_), 5.34 (d, *J* = 2.50, H-4), 5.92 (d, *J* = 2.50, H-3), 5.90 (s, 2H, pyrrole), 6.85 (m, 1H, thiophene), 7.11-8.66 (m, 13H, Ar-H); ^13^C-NMR δ (ppm): 13.42(2C), 62.24, 69.35, 107.79 (2C), 115.42, 120.99, 122.91, 123.74, 124.37, 126.45 (2C), 127.05, 127.21 (2C), 127.51, 127.60, 127.89, 128.03 (2C), 128.79 (2C), 129.31, 130.78, 131.34 (2C), 132.33, 139.10, 148.50, 164.83. Anal. Calcd for C_31_H_24_N_2_OS: C, 78.78; H, 5.12; N, 5.93. Experimental: C, 78.71; H, 5.05; N, 5.90.

## 4. Conclusion

As part of our current research on anticancer β-lactams we have demonstrated an easy preparation of 3-pyrrole-substituted β-lactams through molecular iodine-catalyzed reactions. This simple method may find useful applications in drug synthesis. Furthermore, the compounds reported herein will be tested against a number of cancer cells *in vitro*.
